# Interactive influences of intercropping by nitrogen on flavonoid exudation and nodulation in faba bean

**DOI:** 10.1038/s41598-019-41146-9

**Published:** 2019-03-18

**Authors:** Yingchao Liu, Xinhua Yin, Jingxiu Xiao, Li Tang, Yi Zheng

**Affiliations:** 1 College of Resource and Environmental Science, Yunnan Agricultural University, Kunming, 650201 China; 2 Department of Science and Technology, Kunming University, Kunming, 650214 China; 3 Department of Plant Science, University of Tennessee, Jackson, Tennessee 38301 United States of America; 4 Yunnan Provincial Department of Education, Kunming, Yunan 650223 China

## Abstract

In order to address the question of how flavonoids affected root nodulation of faba bean in a wheat and faba bean intercropping system, we set up soil and hydroponic experiments comprising two cropping pattern treatments (intercropped and monocropped) and three nitrogen (N) supply treatments at the deficient (50% N), adequate (100% N), and excessive (150% N) levels with three replicates in a randomized complete block design. Across the three N treatments and two experiments, it was frequently observed that intercropping increased but N fertilization decreased the nodule number and nodule dry weight of faba bean. Six types of flavonoids were detected in the faba bean root secretion, but only genistein, hesperetin, and naringenin often had significant correlations with the nodule number and nodule dry weight. Intercropping increased faba bean root secretions of genistein, hesperetin, and naringenin compared to monoculture only at the deficient and adequate N supply levels. The differences in flavonoids of faba bean caused by the intercropped patterns, N supply levels, and their interactions were mainly significant at flowering stage. In conclusion, interspecies and N supply interactively altered the contents and proportions of flavonoids in faba bean root exudations under wheat and faba bean intercropping. These findings provide insight into flavonoids-nodule-yield interactions in cereal and legume intercropping systems.

## Introduction

In recent years, agricultural production had to strive for higher yield in order to increase food supply to feed a rapidly growing population. China has become the largest fertilizer producer and consumer in the world^1^. From ammonia, various synthetic nitrogen (N) fertilizers are manufactured, without which, nearly half of the world’s population would not be alive today^1^. However, synthetic N fertilizers have become “too much of a good thing” because a relatively high proportion of the N applied to cropland escapes from the agricultural system and becomes a pollutant, which disrupts terrestrial and aquatic ecosystem functions and contributes to global climate change^2^. Intercropping, as a widely accepted agronomical practice for two thousand years in China, can increase crop yield through increasing resource use efficiencies and improving land-equivalent ratio^3^. Furthermore, if an appropriate combination of crops is chosen for intercropping, it can reduce N fertilizer use and become an environmentally friendly practice^4^.

In conventional agriculture, intercropping of a cereal crop with a grain leguminous crop has been widely used because of their complementarities in the use of N resources^5,6^. Several studies showed substantial complementary use of N in the cereal legume intercropping systems^7–9^. Besides the advantages of using leguminous crops as a fundamental tool for the maintenance of N fertility^10^, there are many other positive effects derived from intercropping legumes with non-leguminous crops, such as enhanced yield stability, smothering of volunteer plants, pathogen and pest reduction, and increased water and nutrient use efficiencies^11–13^. Although faba bean (*Vicia faba L*.) is a crop of relatively minor importance by acreage worldwide, it has been fairly studied during the last two decades due to its high potential in N_2_ fixation^14–16^. However, the wheat-faba bean intercropping system, widely used by farmers especially in southwestern China, has so far not been investigated in detail. Furthermore, there are very few reports revealing the underlying nitrogen-nodules-flavonoids interaction mechanisms in wheat-faba bean intercropping.

Flavonoids are a class of secondary metabolites that exhibit important functions in mediating the interactions between plants and the environment^17^. Flavonoids in root exudates are inducers of genes responsible for the nodulation process of rhizobium in legumes, and are utilized by fungi such as arbuscular mycorrhizae fungi which may be involved in the bacterial inoculation process^18^. In plant-plant interactions, flavonoids are responsible for triggering action and signaling cascades^19–21^ in general response pathways, and are presumably involved in signal transduction after the reception of root exudates *in vitro*^22^. But the effects and related mechanisms on secretion of flavonoids and their influences on legume crop nodulation and N_2_ fixation at different N fertilization levels are so far unclear in leguminous and gramineous intercropping systems. Therefore, the main objectives of the present research were to examine: (1) whether plant interactions in a wheat and faba bean intercropping system could promote flavonoid biosynthesis in the roots of faba bean, and (2) whether low or high N supply levels could enhance root secretions of flavonoids in faba bean, and thus affect its nodulation in a wheat and faba bean intercropping system. Faba bean and wheat as the representative crops of legume and non-legume, respectively, were used to make up an intercropping system in both soil and hydroponic experiments to answer the above questions in this study. It is imperative to recognize the roles performed by roots in a cereal and legume intercropping system and to understand the impacts of plant-plant interactions on rhizospheric processes.

## Results

### Nodulation of faba bean

Intercropping frequently increased root nodulation compared to monoculture across the three N treatments and two experiments (Table 1). On the 95^th^ d in the soil experiment, the nodule number was significantly increased by 60.2%, 80.2%, and 145.2% with intercropped faba bean compared to monocropped faba bean in the deficient, adequate, and excessive N treatments, respectively. In the hydroponic experiment, the nodule number was enhanced by 48.8%, 31.7%, and 93.3% with intercropped faba bean relative to its monoculture under the three N treatments, respectively (Table 1). Nodule dry weight was increased significantly by 1.5, 1.3, and 2.7 times in faba bean under intercropping compared to monocrop in the soil experiment. The nodule dry weight was enhanced significantly by 0.37, 1.33, and 4.5 times in faba bean under intercropping over monocropping at the three N levels, respectively, on the 95^th^ d in the hydroponic experiment (Table 1). On the 131^th^ d, the nodule number of in the soil experiment was increased significantly by 45.2% and 44.1% in intercropped faba bean compared to monocropped faba bean at the deficient and excessive N levels, respectively. The nodule number of the hydroponic experiment was enhanced significantly by 69%, 43.8% and 50% with faba bean under intercropping relative to monocropping at the deficient, adequate, and excessive N levels, respectively (Table 1). The nodule dry weight of faba bean was increased significantly by 1.8, 1.4 and 1.3 times at deficient, adequate, and excessive N under intercropping over monocropping on the 131^th^ d (Table 1) in the soil experiment. However, no difference in nodule dry weight of faba bean was observed between mono- and inter-cropping faba beans under any N treatment in the hydroponic experiment.

**Table 1:** Effects of intercropping and N application on nodule number and nodule dry weight of faba bean.

Experiment	Time	Treatments	Nodule number			Nodule dry weight		
			50% N	100% N	150% N	50% N	100% N	150% N
Soil experiment (g·plant^−1^)	95^th^ d	IF	157^a^ ± 6.24	155^a^ ± 12.90	152^a^ ± 8.54	1.65^a^ ± 0.08	1.12^b^ ± 0.09	0.71^c^ ± 0.05
		MF	98^b^ ± 39.89	86^b^ ± 8.00	62^b^ ± 28.69	0.66^c^ ± 0.12	0.48^d^ ± 0.03	0.19^e^ ± 0.05
	131^th^ d	IF	105^a^ ± 8.89	73^b^ ± 11.36	98^a^ ± 8.19	1.40^a^ ± 0.15	1.15^b^ ± 0.06	0.86^c^ ± 0.13
		MF	72^b^ ± 7.51	69^b^ ± 9.17	68^b^ ± 11.53	0.50^d^ ± 0.05	0.48^d^ ± 0.09	0.37^d^ ± 0.10
Hydroponic experiment (g·plant^−1^)	95^th^ d	IF	119^a^ ± 11.53	79^b^ ± 5.00	58^c^ ± 7.55	0.71^a^ ± 0.12	0.35^c^ ± 0.05	0.33^c^ ± 0.15
		MF	80^b^ ± 5.00	60^c^ ± 6.00	30^d^ ± 5.57	0.52^b^ ± 0.04	0.15^d^ ± 0.04	0.06^d^ ± 0.03
	131^th^ d	IF	71^a^ ± 5.29	57^b^ ± 6.24	46^c^ ± 5.57	0.40^a^ ± 0.21	0.29^a^ ± 0.05	0.28^a^ ± 0.19
		MF	42^cd^ ± 5.29	38^cd^ ± 5.29	32^d^ ± 6.56	0.27^a^ ± 0.05	0.28^a^ ± 0.07	0.22^a^ ± 0.02

IF-intercropped faba bean; MF-monocropped faba bean; different letters denote significant differences among targeted plant data in the wheat//faba bean and single faba bean treatments across different N levels at the same sampling time (p ≤ 0.05).

Increased N fertilization sometimes decreased the nodule number and nodule dry weight of faba bean in both experiments (Table 1). On the 95^th^ d in the soil experiment, no difference in the nodule number was observed among the three N treatments for either inter- or mono-cropping faba beans (Table 1). However, the nodule number of intercropped faba bean was decreased by 105.2% and 36.2% at the excessive N level relative to the deficient and adequate N treatments, respectively, in the hydroponic experiment. The nodule number of monocropped faba bean was reduced by 166.7% and 100% with excessive N compared to deficient and adequate N, respectively, on the 95^th^ d (Table 1). On the 95^th^ d in the soil experiment, the nodule dry weight of intercropped faba bean was significantly reduced by 132.4% and 57.7% and that of monocropped faba bean was decreased by 247.4% and 152.6% at the excessive N level relative to the deficient and adequate N treatments, respectively (Table 1). On the 95^th^ d in the hydroponic experiment, nodule dry weight of intercropped faba bean was reduced by 115.2% at excessive N relative to deficient N, and nodule dry weight of monocropped faba bean was reduced by 7.67 and 1.5 times at excessive N compared to deficient and adequate N, respectively (Table 1). On the 131^th^ d in the hydroponic experiment, the nodule number of intercropped faba bean was decreased by 54.3% and 23.9% with excessive N over deficient and adequate N, respectively (Table 1). On the 131^th^ d in the soil experiment, nodule dry weight of intercropped faba bean was reduced by 62.8% and 33.7% and nodule dry weight of monocropped faba bean was decreased by 35.1% and 29.7% at excessive N compared to deficient and adequate N, respectively (Table 1). However, on the 131^th^ d, almost no significant increment existed in the nodule number of monocropped faba bean in either experiment regardless of N supply level. No significant difference was observed in nodule dry weight of inter- or mono-cropped faba bean in the hydroponic experiment and of monocropped faba bean in the soil experiment among the three N levels (Table 1).

### Correlation between flavonoids and nodulation

A significant linear relationship was frequently observed between the total content of flavonoids and the nodule number or nodule dry weight in the roots at the deficient and adequate N levels in both soil and hydroponic experiments (Table 2). In contrast, no correlation was found between the total content of flavonoids and nodule number at the excessive N level in the soil experiment or between the total content of flavonoids and nodule dry weight under the excessive N condition in either experiment (Table 2). Across all types of flavonoids in both experiments, only genistein, hesperetin, and naringenin had significant correlations with both nodule number and nodule dry weight at some N levels. In the deficient N treatment, these three flavonoids often had significant correlations with both nodule number and nodule dry weight in two experiments (Table 2). Naringenin and hesperetin often had significant linear relationship with the nodule number and nodule dry weight at the deficient and adequate N levels (Table 2). In short, our results suggest both nodule number and nodule dry weight are affected partially by certain types of flavonoids.

**Table 2:** Correlation coefficient of flavonoids with nodule number and nodule dry weight across the 95^th^ and 131^th^ d sampling dates.

Experiment	Index	Treatment	Total Flavonoids	Genistein	Hesperetin	Quercetin	Naringenin	Chalcone	Soy isoflavone
Soil experiment	Nodule number	50% N	0.771**	0.650*	0.609*	0.428	0.797**	0.490	0.372
		100% N	0.922**	0.298	0.750**	0.450	0.792**	0.507	0.528
		150% N	−0.117	0.374	0.368	−0.197	0.351	−0.034	−0.630*
	Nodule dry weight	50% N	0.946**	0.647*	0.778**	0.573	0.886**	0.538	0.575
		100% N	0.617*	0.246	0.523	0.467	0.667*	0.303	0.014
		150% N	0.152	0.172	0.225	0.455	−0.225	0.381	−0.134
Hydroponic experiment	Nodule number	50% N	0.956**	0.661*	0.604*	0.316	0.869**	0.315	0.330
		100% N	0.841**	0.835**	0.798**	0.572	0.596*	0.567	0.565
		150% N	0.741**	0.439	0.170	0.563	0.189	0.378	0.493
	Nodule dry weight	50% N	0.810**	0.506	0.700*	0.443	0.643*	0.300	0.261
		100% N	0.595*	0.258	0.593*	0.084	0.736**	0.465	0.320
		150% N	0.549	0.199	−0.200	0.387	0.576*	0.130	0.421

Asterisks denote significant correlations, n = 12, *p < 0.05; **p < 0.01.

### Root exudation of flavonoids

**Figure 1:**
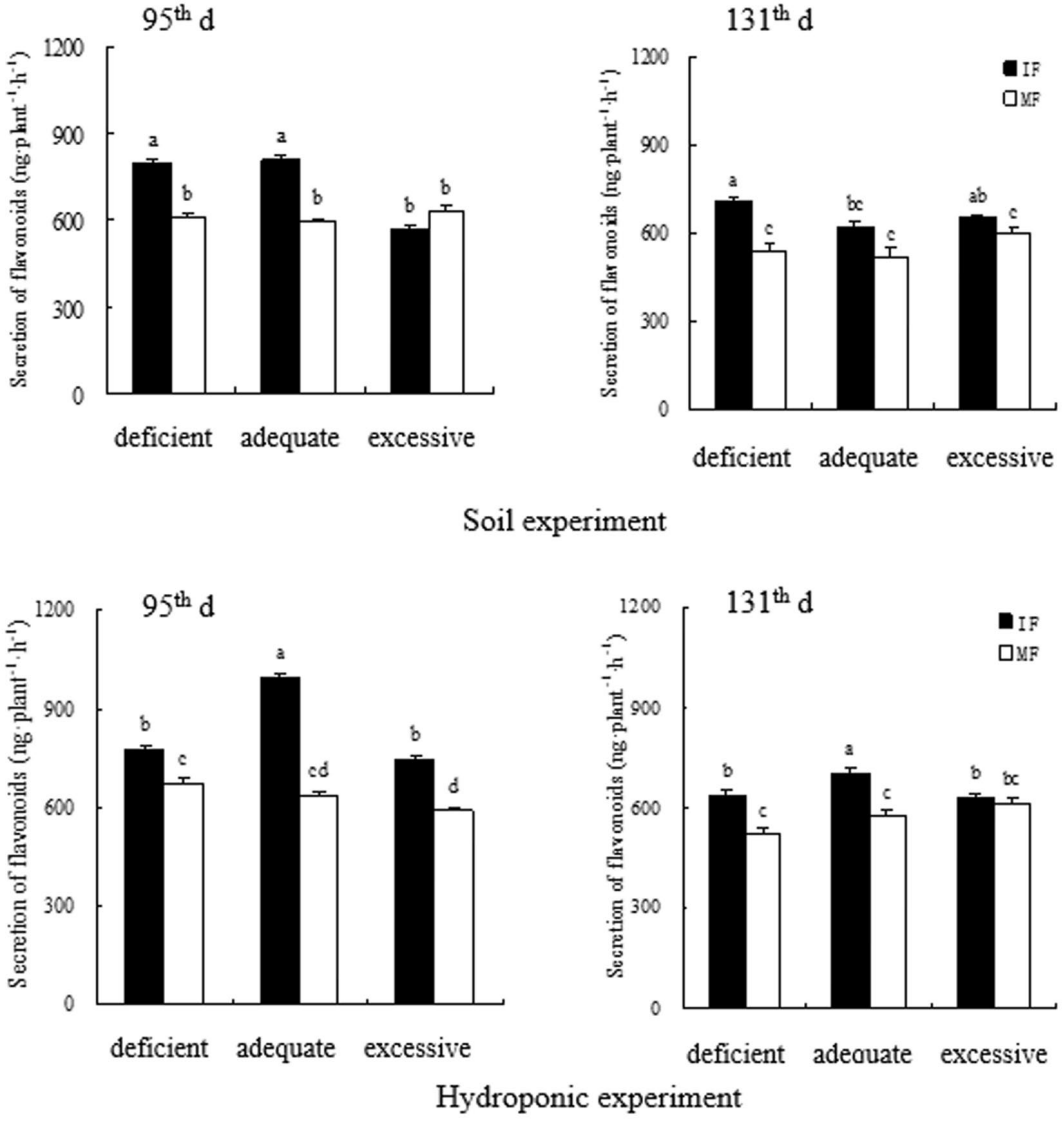
Flavonoids secreted by intercropped and monocropped faba bean under different N supply levels at 95^th^ d and 131^th^ d in both soil and hydroponic experiments.

Faba bean frequently exudated more flavonoids when intercropped with wheat than when grown alone in all N treatments (Fig. 1). On the 95^th^ d, flavonoids secreted by intercropped faba bean roots were increased significantly by 31.7% and 35.6% relative to the monocropping counterpart at the deficient and adequate N levels in the soil experiment and increased significantly by 16.8%, 56.1% and 28.2% in deficient, adequate and excessive N in the hydroponic experiment, respectively (Fig. 1). On the 131^th^ d, flavonoids secreted by intercropped faba bean were enhanced by 29.9% and 15.5% over those by monocropped faba bean in the soil experiment and increased by 22.1% and 24.8% over those by monocropped faba bean in the hydroponic experiment at the deficient and adequate N levels, respectively (Fig. 1).

Increased N application frequently decreased secretion of flavonoids in faba bean roots. On the 95^th^ d in the soil experiment, total flavonoids of intercropped faba bean were decreased by 39.6% and 41% at excessive N relative to deficient and adequate N, respectively; however, the difference in secretion of flavonoids of monocropped faba bean was not significant under any N treatment (Fig. 1). On the 95^th^ d in the hydroponic experiment, total flavonoid of intercropped faba bean were reduced by 31.7% at excessive N relative to adequate N; total flavonoid of monocropped faba bean was decreased by 13.2% with excessive N relative to deficient N (Fig. 1). On the 131^th^ d, total flavonoid of intercropped faba bean was decreased by 17.1% under adequate N compared to deficient N in the soil experiment and reduced by 11.6% at excessive N over adequate N in the hydroponic experiment (Fig. 1). The differences in secretion of flavonoids by monocropped faba bean were not significant among the three N treatments in either experiment (Fig. 1).

**Figure 2:**
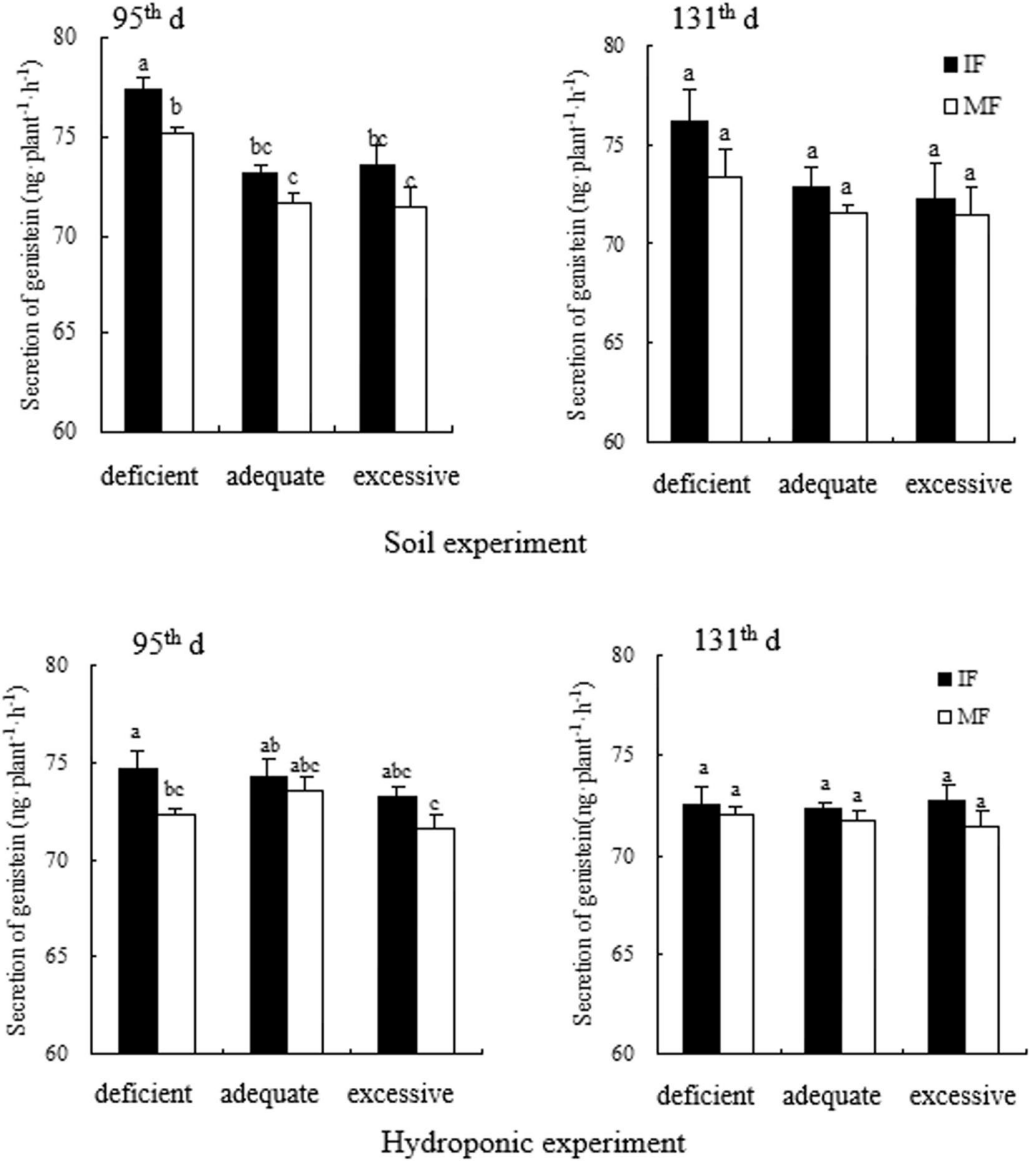
Genistein secreted by intercropped and monocropped faba bean under different N supply levels at 95^th^ d and 131^th^ d in both soil and hydroponic experiments.

**Figure 3:**
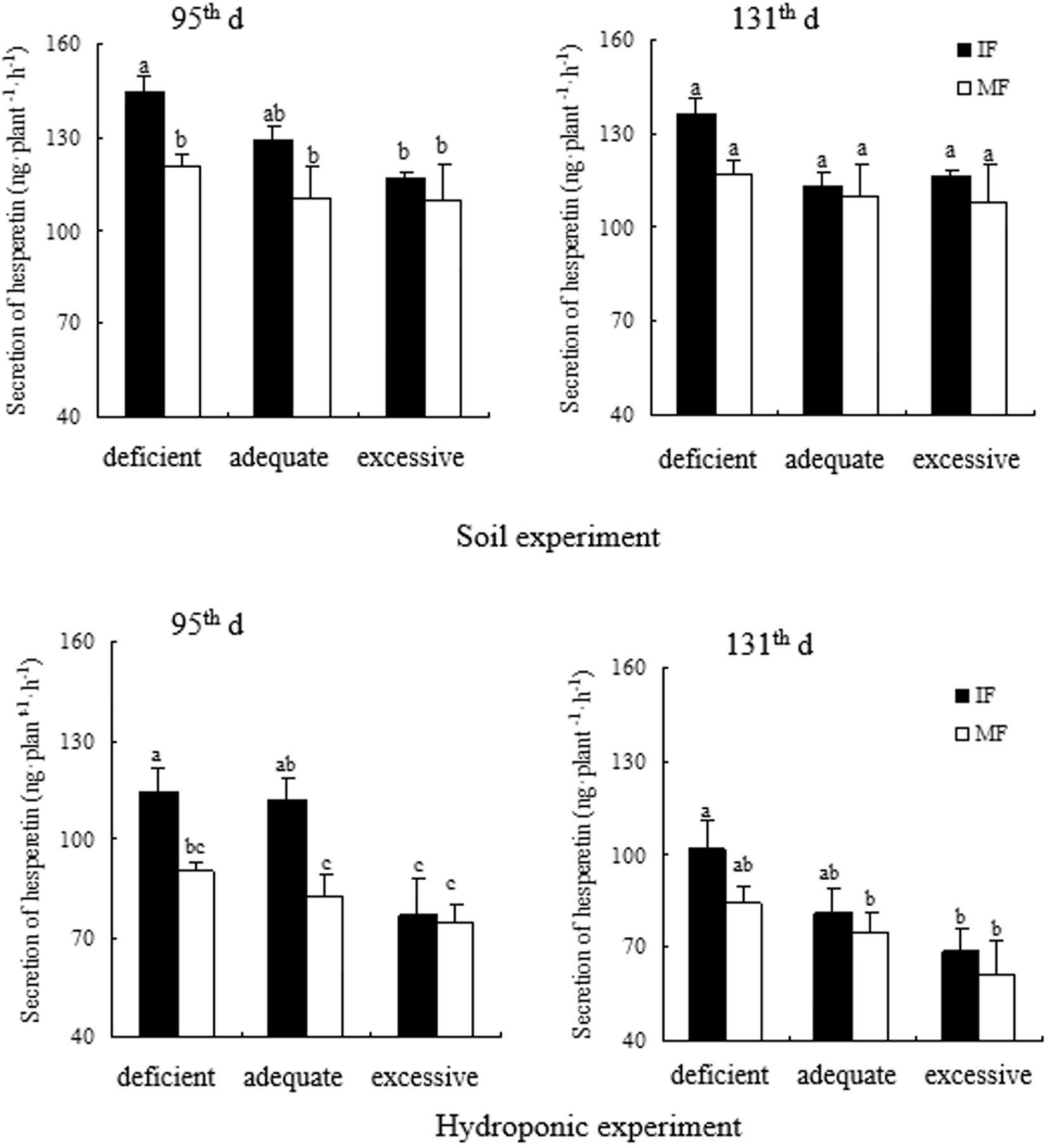
Hesperetin secreted by intercropped and monocropped faba bean under different N supply levels at 95^th^ d and 131^th^ d in both soil and hydroponic experiments.

**Figure 4:**
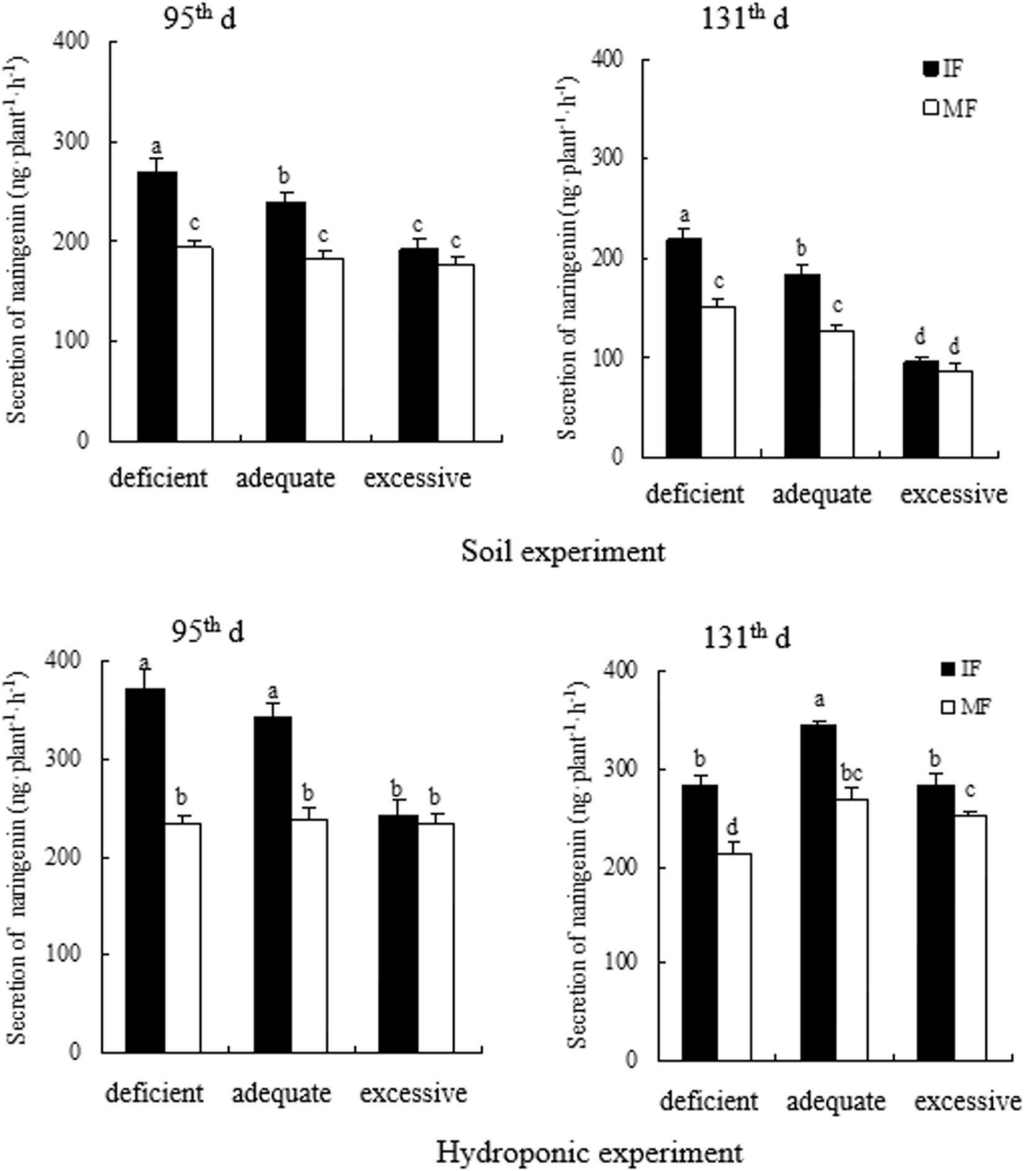
Naringenin secreted by intercropped and monocropped faba bean under different N supply levels at 95^th^ d and 131^th^ d in both soil and hydroponic experiments.

Across the three N treatments and two experiments, intercropped faba bean increased secretion of genistein at deficient N and hesperetin and naringenin at the deficient and adequate N levels relative to its monoculture on the 95^th^ d. On the 95^th^ d, genistein of intercropped faba bean under the deficient N condition was increased by 3.0% in the soil experiment and 3.4% in the hydroponic experiment compared to that in monocropping (Fig. 2). In the soil experiment, hesperetin of intercropped faba bean was enhanced by 19.9% at deficient N and naringenin of intercropped faba bean was increased by 39.1% and 28.9% at the deficient and adequate N levels, respectively, over that of monocropped faba bean on the 95^th^ d (Figs 3 and 4). In the hydroponic experiment, hesperetin was increased by 26.9% and 35.6% and naringenin was enhanced by 58.8% and 43.8% with intercropped faba bean relative to monocropped faba bean at the deficient and adequate N levels, respectively, on the 95^th^ d (Figs 3 and 4). On the 131^th^ d, genistein and hesperetin of faba bean intercropped with wheat were not significantly different from those of faba bean in monoculture at any N supply level in either experiment. However, naringenin of intercropped faba bean was increased by 45.1% and 42.9% in the soil experiment and 32.9% and 17.8% in the hydroponic experiment compared to that of the monocropping counterpart under the deficient and adequate N conditions, respectively (Figs 2–4).

Across the two cropping patterns and two experiments, increased N application sometimes changed the secretion of flavonoids in faba bean roots (Figs 2–4). On the 95^th^ d, genistein of intercropped faba bean was decreased by 5.3% at excessive N relative to deficient N in the soil experiment; however, the difference in secretion of genistein was not significant among the N treatments in the hydroponic experiment (Fig. 2). Hesperetin of intercropped faba bean with excessive N was reduced by 23.6% relative to intercropped faba bean at deficient N in the soil experiment, and decreased by 48.3% and 44.4% compared to intercropped faba bean at deficient and adequate N, respectively, in the hydroponic experiment on the 95^th^ d (Fig. 3). Correspondingly, naringenin of intercropped faba bean with excessive N was reduced by 48% and 28.5% in the soil experiment and decreased by 52.7% and 41.2% in the hydroponic experiment, relative to the deficient and adequate N treatments, respectively, on the 95^th^ d (Fig. 4). Except that genistein of monocropped faba bean was reduced by 5.1% at excessive N relative to deficient N in the soil experiment, other flavonoids of monocropped faba bean were not different among the three N levels on the 95^th^ d in either experiment (Figs 2–4). On the 131^th^ d across the two experiments, no difference in genistein was observed among the three N concentrations in either inter- or mono-cropping faba bean (Fig. 2). On the 131^th^ d, hesperetin of intercropped faba bean was decreased by 48.1% under excessive N relative to deficient N in the hydroponic experiment, but the differences of intercropped faba bean were not significant among the three N treatments in the soil experiment (Fig. 3). Naringenin of intercropped faba bean was reduced by 129.4% and 92.3% with excessive N relative to deficient and adequate N, respectively, on the 131^th^ d in the soil experiment. In the hydroponic experiment, naringenin of intercropped faba bean was reduced by 12.1% under excessive N relative to adequate N on the 131^th^ d (Fig. 4). Naringenin of monocropped faba bean was decreased by 75.9% and 49.8% at excessive N compared to deficient and adequate N, respectively, on the 131^th^ d in the soil experiment. There was no significant difference among the three N treatments on the 131^th^ d in hesperetin of monocropped faba bean roots in either experiment or in naringenin of monocropped faba bean in the hydroponic experiment(Figs 3 and 4).

## Discussion

The method for collection and identification of flavonoids from the root system in this study was common to those used in previous investigations^23^ as it is easy to operate. However, it had some limitations. For instance, root exudations collected in the hydroponic culture medium might not be the same as those released by plants grown in the soil. Although a continuous root exudate trapping system was used to overcome the difficulties in sample collection, it still couldn’t be widely applied because of contamination, generation of inhibitor, microbial toxins, and so on^24^. Thus, a new *in situ* method for collection of root exudations should be developed in the future. Furthermore, GC-MS and HPLC are widely used for quantitative and qualitative identification of root exudations, and several flavonoids have been identified in plants with these methods^25^. In the present study, we only quantitatively identified six major flavonoids in root exudations. Combining these two methods together would be beneficial for understanding the characteristics of flavonoids and the mechanism of increased flavonoid accumulation in promoting N_2_ fixation through nodule formation in the intercropping systems.

Intercropping systems improved root nodulation and N_2_ fixation in leguminous crops and led to yield increases^13,26^. Intercropped faba bean increased the nodule number by 13.92 and 11.97 per plant at the flowering and pod stages, respectively, and enhanced yield by 22.3% over monocropped faba bean^26^. Intercropping systems increased nodulation as N application level decreased^23^. Nodulation of faba bean intercropped with maize was increased by 115% with no N supplied and 95% at a high N application rate over that of monocropped faba bean^23^. Our results showed that the intercropping system increased faba bean nodule number and nodule dry weight in both soil and hydroponic experiments. Compared to those of monocropped faba bean at the flowering stage (sampled at the 95^th^ d), the nodule number of intercropped faba bean was increased by 60.2%–145.2% in the soil experiment and 31.7%–93.3% in the hydroponic experiment. Similarly, nodule dry weight was increased significantly by 1.3–2.7 times in the soil experiment and 36.5%–4.5 times in the hydroponic experiment, relative to those of monocropped faba bean at the flowering stage. However, these advantages under intercropping gradually became less obvious as faba bean continued to develop (sampled at the 131^th^ d), which indicated that the intercropping system stimulated root nodulation and N_2_ fixation, but that effect decreased with time. Nitrogen affected nodulation of leguminous crops in gramineous and legume intercropping systems. Under monoculture conditions, N exerted “N repression” effect which led to the inhibition of nodulation and N_2_ fixation in legumes;^27^ however, intercropping could slow down the effect of “N repression”, and that effect was regulated by the N application rate^28^.

Plants may release large amounts of root exudations^29^, which provides carbon for bacteria or act as signal molecules in the root-root interactions among plant species^30^. Flavonoids as secondary metabolites in root exudations played a crucial role in promoting the formation of nodules by symbiotic bacteria which is commonly known as rhizobia^31^. Therefore, we investigated the flavonoid effects on nodules in the faba bean and wheat intercropping system. Several flavonoids including naringenin, hespertin, genistein, quercetin, chalcone, and soy isoflavone were identified in faba bean roots. In our experiments, only naringenin, hespertin, and genistein frequently had a significant linear relationship with the nodule number and nodule dry weight. Therefore, the increased nodulation in faba bean intercropped with wheat was likely caused by the change in naringenin, hespertin, and genistein in root exudates.

Our results (Table 2; Figs 1–4) indicated that secretion of flavonoids by faba bean roots was sometimes affected by the cropping pattern and N supply level. Intercropped pattern rarely influenced the secretion of genistein in faba bean at the deficient N level, but increased the secretion of hesperetin and naringenin at the deficient and adequate N levels on the 95^th^ d. Hesperetin was increased by 19.9% under the adequate N condition and naringenin was increased by 28.9%–39.1% in the deficient and adequate N treatments in intercropped faba bean relative to monocropped faba bean in the soil experiment. Hesperetin was enhanced by 26.9%–35.6% and naringenin was increased by 43.8%–58.8% in intercropped faba bean compared to monocropped faba bean in the hydroponic experiment. However, these three types of flavonoids of faba bean intercropped with wheat were not significantly different from those of faba bean in monoculture on the 131^th^ d. Faba bean under both cropping patterns secreted more flavonoids at the flowering stage (95^th^ d) than the other growth stages in our study. These findings agree with those of previous studies^32,33^. Nitrogen had significant effects on the composition and contents of flavonoids secreted by the legume roots^34,35^. However, the N effect decreased as the N application rate increased. This trend was possibly caused by the low competing capacity of legume compared to cereal for mineral N^36,37^. At the deficient N level, wheat intercropped with faba bean secured more N from faba bean because of wheat’s greater competing capacity for mineral N^38,39^. In order to promote nodulation and N_2_ fixation, legume accumulated a much larger amount of flavonoids under intercropping than monocropping. This effect led to enhanced wheat growth and yield^40^. However, this effect mostly disappeared at the adequate and excessive N levels. When adequate N nutrition was supplied, the competition for N became less intense between faba bean and wheat and faba bean did not need to fix N_2_, so its nodule number and nodule dry weight decreased as flavonoid concentration went down^41^. Genistein as a nod gene inducer in pre-incubation of *Bradyrhizobium japonicum* accelerated the development of nodules at low temperatures^42,43^. The secretion of genistein by the roots was similar from maize or faba bean but more from barely or wheat^44^. Maybe the secretion of genistein caused faba bean intercropped with maize to increase the nodule number over monocropped faba bean under no N application^45^. However, there was no significant difference in genistein at any N level in either experiment in this study. This indicated that the N supply level had less effect on genistein than the cropping pattern, which might be resultant from the other flavonoids which had effects on genistein. A positive effect of naringenin on nodulation was reported in an alfalfa-*R.meliloti* system^46^, which acted as a suppressor of nod genes of a standard strain of this bacterium^47,48^. In pea, a rhizospheric application of naringenin partially alleviated the negative effect of low temperature on nodulation^49^. Similar inhibiting effect of hesperetin on nodule formation was also observed at the highest concentrations tested^50^. Maybe naringenin and hesperetin in the root system both have significant inhibiting effects on the accumulation of genistein. Genistein within a certain concentration range maybe is the core material for nodulation of legume plants, and naringenin and hesperetin in the rhizosphere keep the concentration of genistein within an appropriate range for the nodulation process.

## Conclusions

Faba bean intercropped with wheat resulted in improved root nodulation and N_2_ fixation than faba bean in monoculture. Increased N fertilization rate decreased the nodule number, nodule dry weight, and flavonoid secretion of faba bean. Furthermore, intercropped faba bean increased the root secretion of hesperetin, naringenin, and genistein relative to faba bean in monoculture only at the lower N supply levels including deficient (50% N) and adequate (100% N). This suggests intercropping and N supply interactively alter the contents and proportions of flavonoids in faba bean root exudations under the wheat and faba bean intercropping. Across all flavonoids detected in the two experiments, only naringenin, hesperetin, and genistein had linear relationship with nodule index, but the mechanism was not clear for the effects of these three flavonoids on nodulation. These results could partially explain why intercropping increased secretion of flavonoids and nodulation of faba bean. In order to promote root nodulation and N_2_ fixation, legume accumulated much higher amount of flavonoids in intercropping than monocropping. Increased flavonoid accumulation promoted N_2_ fixation through nodule formation in the intercropping system. It is imperative to verify the outcomes of this study by conducting an exogenous addition of flavonoids test in the future.

## Materials and Methods

### Experimental set-up

#### Soil experiment

A soil experiment was conducted with two cropping patterns and three N supply levels. Faba bean was grown alone (monocropped/single faba bean), and intercropped with wheat (faba bean//wheat, root separated with nylon) in the soil supplied with three N fertilization levels at 75, 150, and 225 mg N kg^−1^ of soil, which represented deficient (50% N), adequate (100% N) and excessive (150% N) treatments, respectively. There were six treatment combinations in total arranged in a randomized complete block design with three replicates per treatment.

The pot size was 338 mm (diameter) × 351 mm (height), and each pot was filled with 10 kg of soil. The soil was collected from the Yunnan Agricultural University Experimental Station in Kunming, China, aired-dried, passed through a 5-mm sieve, and thoroughly mixed before potting. Initial soil properties were determined, and the results are presented as follows: Available N: 146.5 mg kg^−1^, Olsen-P: 55.6 mg kg^−1^, Available K: 169.2 mg kg^−1^, Organic C: 49.2 mg kg^−1^, and pH: 6.08^51^. Urea was applied as the N fertilizer in this study.

The genotype of wheat (*Triticum aestivum L*.) Yunmai-47 and the genotype of faba bean (*Vicia faba L*.) Yundou-8363 were used in this study. Uniform wheat and faba bean seeds were sterilized in 30% v/v H_2_O_2_ solution for 20 min, washed with deionized water, soaked in CaSO_4_ saturated solution for 12 h, and then germinated at 22 °C in petri dishes covered with wet filter papers for 1–2 d. Twelve germinated faba bean seeds were planted in two rows in each pot in the monocropped faba bean treatment; for the wheat and faba bean intercropping system, however, a half space of pot was planted with fourteen germinated wheat seeds, and the other half space was planted with six germinated faba bean seeds in each pot. All pots were randomly arranged and rotated among the treatments on a weekly basis during the experimentation. The plants were watered every day to maintain field capacity moisture of the soil (18%-20%, w/w).

Plants were sampled for counting the number of root nodules and collecting root secretion after 60 (seeding stage), 95 (flowering stage), 131 (podding stage), 157 (grain filling stage), and 181 d (maturity stage) of sowing. Following root excavation, plants were gently shaken to dislodge the rhizospheric soil, and then washed with deionized water for three times. Roots were then transferred to a tube containing 500 ml of 0.2 mm CaCl_2_ solution with 5% concentration thyme camphor for 2 hours to generate flavonoid suspensions. After the suspensions were extracted with 200, 100, and 50 ml ethyl acetate separately, the extracted root exudate collection was evaporated to 10 ml under reduced pressure at 40 °C on a rotary evaporator. A volume of 1 ml titrating solution was added with a pipette to a 10-ml sample bottle after passing through 0.45 μm filter membrane for flavonoid analysis with high performance liquid chromatography (HPLC).

#### Hydroponic experiment

A hydroponic experiment was set up with two cropping pattern treatments (wheat//faba bean, intercropped crop roots separated with nylon, and monocropped faba bean) interacted with three N supply (deficient-50% N, adequate-100% N, and excessive-150% N) treatments, totaling six treatments arranged in a randomized complete block design with three replicates of each treatment.

The same genotypes of wheat and faba bean seeds were chosen and handled as what was done in the soil experiment, and the N supply levels were selected based on a preliminary experiment with the same nutrient solution except N. In order to ensure adequate nutrient supply for plant growth, nutrient solution was made at the following rates (mmol nutrient product per L of solution): K_2_SO_4_ 0.75; MgSO_4_ 0.65; KCl 0.1; KH_2_PO_4_ 0.25; H_3_BO_3_ 0.01; MnSO_4_ 0.001; CuSO_4_ 0.0001; ZnSO_4_ 0.001; (NH_4_)_6_Mo_7_O_24_ 0.005; and Fe-EDTA 0.1. After seeds were germinated with two expanded cotyledons; the roots of seedlings were watered, and the seedlings were then transplanted into the pots filled with 3 L of the sterilized nutrient solution. The pH of nutrient solution was maintained at approximately 6.0 during the experimentation.

The concentration of nutrient solution was diluted to 1/4 on the 7th day after transplanting and 1/2 on the 15th day after transplanting, but full concentration of the nutrient solution was maintained from the 30th day after transplanting till maturity. There were 2 faba bean and 4 wheat plants in intercropping and 4 plants under monocropped faba bean in each pot. The nutrient solution was replaced once every three days. Nitrogen was supplied as Ca(NO_3_)_2_. The adequate N supply rate was 2.0 mmol per L; 50% N represented half of the adequate N rate, 100% N represented the adequate N rate, and 150% N was 1.5 times of the adequate N rate, representing the excessive N apply. Oxygen was added into the sterilized nutrient solution continuously day and night.

Plants were sampled from the pots. The roots were flushed repeatedly for three times with tap water, rinsed with distilled water, and then transferred into a tube containing 500 ml of 0.2 mmol per L CaCl_2_ solution with 5% concentration thyme camphor. The root and solution mixture was maintained for 2 hours to generate a flavonoid suspension. All other sampling and measuring procedures including the collection of root secretions were identical as those used in the soil experiment.

Both experiments were conducted in a greenhouse at Yunnan Agricultural University, Kunming (latitude: 25°02′11″ N, longitude: 102°42′31″ E), China. In the soil experiment, the temperature in the greenhouse was maintained at 21–25 °C during the day and 12–15 °C at night with 12–14 h daytime throughout the experimental period. In the hydroponic experiment, temperature was maintained at 24–28 °C during the day and 15–18 °C at night with 14–19 h day time.

### Measurements

#### Nodulation

The nodule number was counted after the plants were washed. The nodules were separated with tweezers and then were placed into an oven at 70 °C. After the nodules were completely dry, their dry weight was determined.

#### Flavonoids

**Figure 5:**
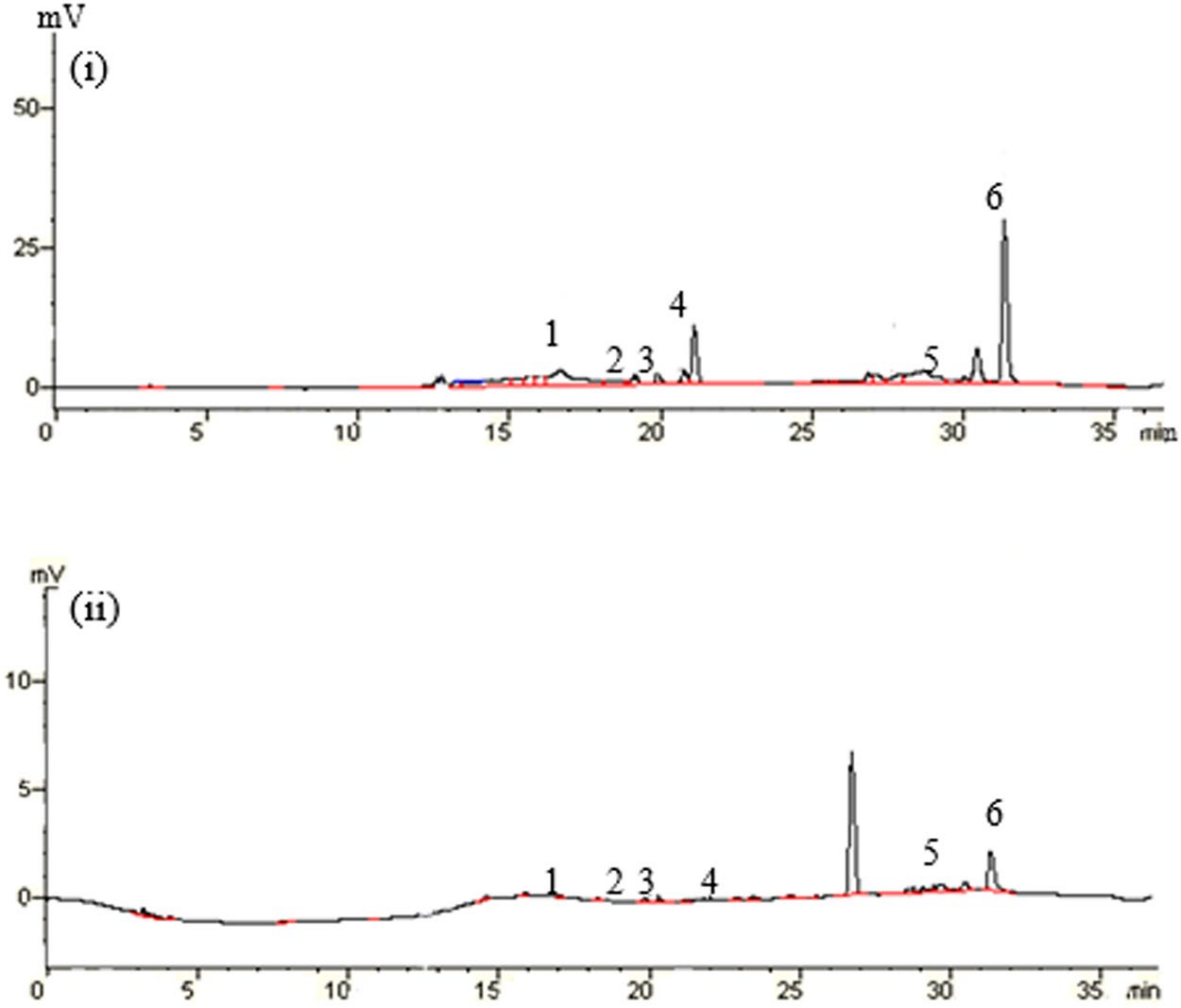
Chromatograms of flavonoids.

Flavonoids were analyzed using a reversed phase high performance liquid chromatography (HPLC) system according to a previous report^23^. The chromatographic separation was conducted on a 250 × 4.6 mm reversed-phase column (Alltima C_18_, 5 Micrometers; Alltech Associates LNC, Deerfield, IL, USA). Solvent A consisted of 99.5% ultra-pure water and 0.5% acetic acid, and solvent B comprised 100% chromatographic pure methanol. The following gradients were used: 30–40% B (5 min); 40–60% B (5 min); 60–90% B (15 min); 90% B (4 min); 90–30% B (5 min); 30% B (3 min); together 37 min with a flow rate of 0.9 ml min^−1^ at 30 °C. Detection of flavonoids was carried out at 270 nm. The separations of six standard flavonoids in the root exudation samples are shown in Fig. 5.

### Statistical analysis

Analysis of variance (ANOVA) was conducted using the SPSS statistical software (SPSS version 19.0, IBM SPSS Inc., Chicago, IL, USA). The treatments were treated as a fixed factor, but the replicates were treated as a random factor. The treatment means were separated using one-way analysis of variance for the cropping pattern by N level treatments with the protected Duncan’s multiple range test at the p ≤ 0.05 probability level.

## Data Availability

The dataset generated in the present study is available from the corresponding author on a reasonable request.
